# Draft Genome Sequence of *Streptomyces* sp. Strain NL15-2K, a Degrader of Lignin-Derived Aromatic Compounds, Isolated from Forest Soil

**DOI:** 10.1128/MRA.01456-18

**Published:** 2019-03-07

**Authors:** Motohiro Nishimura, Susumu Kawakami, Hideaki Otsuka

**Affiliations:** a Department of Pharmaceutical Chemistry, Faculty of Pharmacy, Yasuda Women’s University, Hiroshima, Japan; University of Maryland School of Medicine

## Abstract

Streptomyces sp. strain NL15-2K is a degrader of lignin-derived aromatic compounds and was isolated from a forest soil sample. Here, we report the draft genome sequence of this strain and its annotation. This genome of 12,072,023 bp exhibits a GC content of 70.32% and encodes 10,874 predicted proteins and 75 RNAs.

## Abstract (precis)

Streptomyces sp. strain NL15-2K is a degrader of lignin-derived aromatic compounds and was isolated from a forest soil sample.

## ANNOUNCEMENT

Most bacteria of the genus Streptomyces are found in the soil, where they play a critical role in the global carbon cycle (1). This role can be implemented because streptomycetes have evolved complex and efficient enzymatic systems that catabolize diverse organic substances, such as lignin and lignin-derived aromatic compounds (2, 3). Therefore, Streptomyces species and their enzymes are promising as biocatalysts in the production of commercially valuable compounds, such as vanillin, from inexpensive plant constituents (4). Strain NL15-2K was isolated from a forest soil sample on the campus of the University of British Columbia, Vancouver, Canada, by screening for bacteria capable of catabolizing lignin-derived aromatic compounds (5). This strain was identified as a Streptomyces species by 16S rRNA gene analysis (5).

Streptomyces sp. strain NL15-2K was cultivated for 2 days in yeast extract-malt extract (YEME) medium (6) supplemented with 17% sucrose and 0.5% glycine at 30°C. Genomic DNA was extracted and purified using the Genomic-tip 100/G kit (Qiagen), according to the manufacturer’s protocol. DNA library preparation (paired-end 2 × 100-bp reads) and sequencing were performed on the Illumina HiSeq 2500 (CASAVA version 1.8.2) sequencing platform by Hokkaido System Science Co., Ltd. (Hokkaido, Japan). Shotgun sequencing generated 24,114,726 high-quality paired-end reads. All reads were cleaned up using cutadapt version 1.1 (7) and Trimmomatic version 0.32 (8) by trimming adapter sequences and removing low-quality reads, respectively. The resulting 22,713,654 reads with a mean size of 98 bp were assembled into the genome sequence using Velvet version 1.2.08 (9), and the gaps were closed using Platanus version 1.2.4 (10). Scaffolding was performed using MeDuSa version 1.6 (11), with the Streptomyces lincolnensis NRRL 2936 genome (GenBank accession number CP016438) used as a guide for alignment. The draft genome was annotated using the RAST server (http://rast.nmpdr.org/) (12), with an additional annotation being conducted using antiSMASH version 4.1.0 (13).

The genome size of Streptomyces sp. NL15-2K was 12,072,023 bp, with a GC content of 70.3%, and it comprised 292 scaffolds with an *N*_50_ value of 103,610 bp. Gene prediction and annotation revealed that the Streptomyces sp. NL15-2K genome comprises 10,874 protein-coding sequences and 75 RNA-coding sequences, including 4 rRNAs and 71 tRNAs. The antiSMASH algorithm predicted gene clusters for the biosynthesis of coelichelin, alkylresorcinol, gamma-butyrolactone, albaflavenone, desferrioxamine B, ectoine, endophenazines, indigoidine, and GE37468. Moreover, the genome contained genes involved in the catabolism of lignin-derived aromatic compounds, including *pcaHG* (14) and *catA* (15), which encode protocatechuate 3,4-dioxygenase and catechol 1,2-dioxygenase, respectively, and play a role in cleavage of the aromatic ring. Thus, this study provides valuable genetic information required to understand the catabolism of lignin-derived aromatic compounds in strain NL15-2K and to develop biocatalysts for producing valuable compounds from inexpensive plant constituents.

### Data availability.

The draft genome sequence of Streptomyces sp. NL15-2K has been deposited in DDBJ/ENA/GenBank under accession numbers BHXA01000001 to BHXA01000292. The raw sequencing reads have been submitted to the DDBJ/Sequence Read Archive under the accession number DRA007948.
